# An overview of brain-like computing: Architecture, applications, and future trends

**DOI:** 10.3389/fnbot.2022.1041108

**Published:** 2022-11-24

**Authors:** Wei Ou, Shitao Xiao, Chengyu Zhu, Wenbao Han, Qionglu Zhang

**Affiliations:** ^1^The School of Cyberspace Security, Hainan University, Hainan, China; ^2^Henan Key Laboratory of Network Cryptography Technology, Zhengzhou, China; ^3^The School of Computer Science and Technology, Hainan, China; ^4^State Key Laboratory of Information Security, Institute of Information Engineering, Chinese Academy of Sciences, Beijing, China

**Keywords:** brain-like computing, neuronal models, spiking neuron networks, spiking neural learning, learning algorithms, neuromorphic chips

## Abstract

With the development of technology, Moore's law will come to an end, and scientists are trying to find a new way out in brain-like computing. But we still know very little about how the brain works. At the present stage of research, brain-like models are all structured to mimic the brain in order to achieve some of the brain's functions, and then continue to improve the theories and models. This article summarizes the important progress and status of brain-like computing, summarizes the generally accepted and feasible brain-like computing models, introduces, analyzes, and compares the more mature brain-like computing chips, outlines the attempts and challenges of brain-like computing applications at this stage, and looks forward to the future development of brain-like computing. It is hoped that the summarized results will help relevant researchers and practitioners to quickly grasp the research progress in the field of brain-like computing and acquire the application methods and related knowledge in this field.

## Introduction

Achieving artificial intelligence as the major goal of mankind has been at the top of the heated debate. Since the Dartmouth Conference in 1956 (McCarthy et al., 2006), the development of AI has gone through three waves. They can be roughly divided into four basic ideas: symbolism, connectionism, behaviorism, and statism. These different ideas have captured some of the characteristics of “intelligence” in different aspects, but only partially surpassed the brain of humans in the aspect of function. In recent years, the computer hardware base has become more perfect, and deep learning has revealed its huge potential (Huang Y. et al., 2022; Yang et al., 2022). In 2016, AlphaGo defeated Lee Sedol, the ninth-degree Go master, which marked that the third wave of artificial intelligence technology revolution has reached its peak.

In particular, the realization of AI has become one of the wrestling points of national power competition. In 2017, China released and implemented a new generation of artificial intelligence development planning. In June 2019, the United States released the latest version of the *National Artificial Intelligence Research and Development Strategic Plan* (Amundson et al., 1911). Europe has also identified AI as a priority development project: in 2016, the European Commission proposed a legislative motion on AI; in 2018, the European Commission submitted the *European Artificial Intelligence* (Delponte and Tamburrini, 2018), and published *Coordinated Plan on Artificial Intelligence* with the theme “Made in Europe with Artificial Intelligence.”

Achieving artificial intelligence requires more powerful information processing capabilities, but relying on the current classical computer architecture cannot meet the huge amount of data computing. The classical computer system has encountered two major bottlenecks in its development: the storage wall effect due to von Neumann structure and Moore's law will fail in the next few years. On the one hand, traditional processor architecture is inefficient and energy intensive. When dealing with intelligent problems in real-time, it is impossible to construct suitable algorithms for processing unstructured information. In addition, the mismatch between the rate of programs or data transferred back and forth and the rate of the central processor processing information leads to a storage wall effect. On the other hand, as the chip's size assembly gets closer to the size of a single atom, the devices are getting closer to the limits of their respective physical miniaturization. So, the cost of performance enhancement will become higher and the technical implementation will become more difficult. Therefore, researchers put their hopes on brain-like computing in order to break through the current technical bottleneck.

Early research in brain-like computing followed the traditional computer manufacturing process that we first recognize how the human brain works and develop a neuromorphic computer based on the theory. But after more than a decade of research, mankind is almost standing still in the field of brain science. So, the path of theory before technology was abandoned by mainstream brain-like research. Looking back at human development, we see that many technologies precede theories. For example, in the case of airplanes, we can build the physical object before conducting research to refine the theory. Based on it, researchers adopted structural brain analogs: using existing brain science knowledge and technology to simulate the structure of the human brain, and then refining the theory after success.

**Figure 1:**
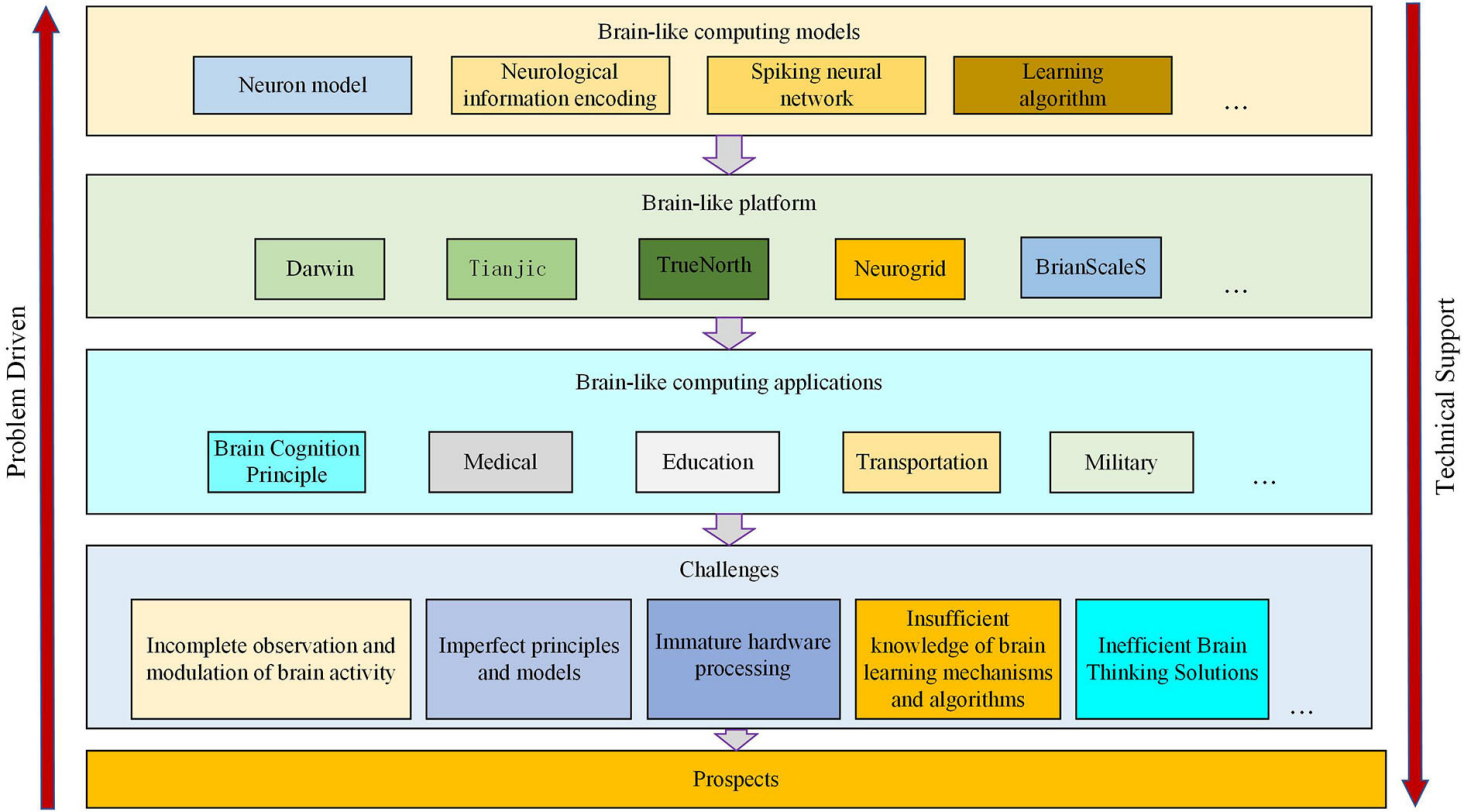
The structure of the article is as follows the analysis of relevant models, the establishment of related platforms, implementation of related applications, challenges, and prospects.

This article first introduces the idea behind the research significance of brain-like computing in a general way. Then we summarize the research history and compare the current research progress with analysis and outlook. The article structure is shown in Figure 1.

## Progress in brain-like computing

Brain-like computers use spiking neural networks (SNNs) instead of the von Neumann architecture of classical computers and use micro and nano-optoelectronic devices to simulate the characteristics of information processing of biological neurons and synapses (Huang, 2016). Brain-like computers are not a new idea, in 1943, before the invention of the computer, Turing and Shannon had a debate about the imaginary “computer” (Hodges and Turing, 1992). In 1950, Turing mentioned it in Computers and Intelligence (Neuman, 1958). In 1958, Von Neumann also discusses neurons, neural impulses, neural networks, and information processing mechanisms of the brain of humans in the Computers and the Human Brain (Yon Neumann, 1958). However, due to the limitations of various technologies at that time and the ideal future described by Moore's theorem, brain-like computing did not receive enough attention. Around 2005, it was generally believed that Moore's law would come to an end around 2020. Researchers began to shift their focus to brain-like computing. Then, the brain-like computing officially entered an accelerated period of development.

**Figure 2:**
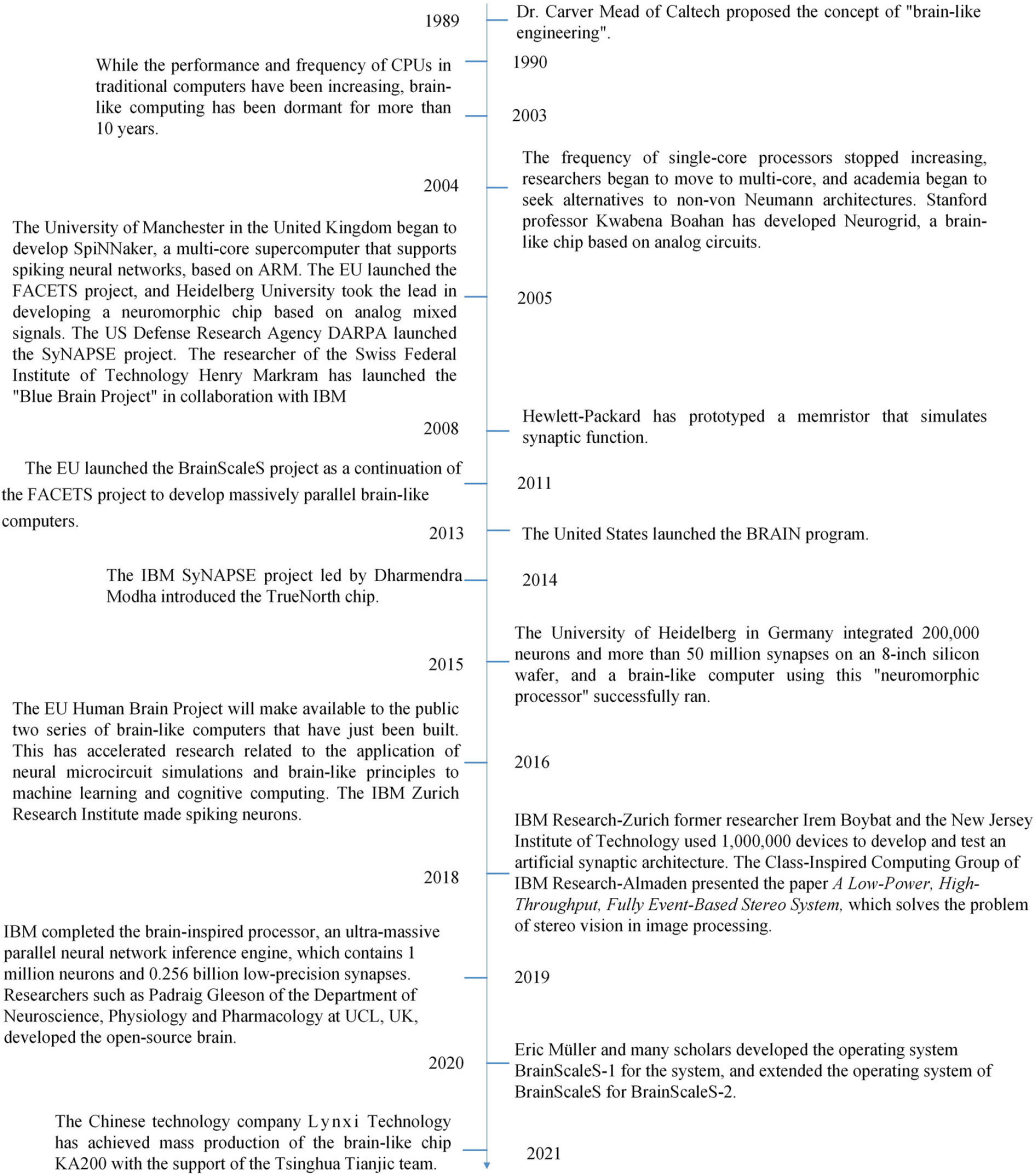
Brain-like computing has evolved from conceptual advancement to technical hibernation to accelerated development due to the possible end of Moore's law.

A summary of the evolution of brain-like computing (Mead, 1989; Gu and Pan, 2015; Andreopoulos et al., 2018; Boybat et al., 2018; Gleeson et al., 2019) is shown in Figure 2.

## Brain-like computing models

There are three main aspects of brain-like computing: simulation of neurons, information encoding of neural systems, and learning algorithms of neural networks.

### Neuron model

Neurons are the basic structural and functional units of the human brain nervous system. The most commonly used models in the SNN network construction are the Hodgkin–Huxley (HH) model (Burkitt, 2006), integrate-and-fire (IF) model (Abbott, 1999; Burkitt, 2006), leaky integrate-and-fire (LIF) model (Gerstner and Kistler, 2002), Izhikevich model (Izhikevich, 2003; Valadez-Godínez et al., 2020), and AdExIF model (Brette and Gerstner, 2005), and so on.

1) HH model

The HH model is closest to biological reality in the description of neuronal features and is widely used in the field of computational neuroscience. It can simulate many neuronal functions, like activation, inactivation, action potentials, and ion channels. The HH model describes the neuronal electrical activity in terms of ionic activity. The cell membrane contains sodium, potassium, and leaky channels. Each ion channel has different gating proteins. It can restrict the passage of ions, so the permeability of each kind of ions is different in the membrane. Because of this, neurons have abundant electrical activity. At a mathematical level, the binding effect of gating proteins is equivalent to ion channel conductance. The conductance of the ion channel, as a dependent variable, varies with the variables of activation and deactivation of the ion channel. The current of the ion channel is determined by the conductance of ion channel, the reversal potential of the ion channel, and the membrane potential. And the total current consists of the leakage, sodium, potassium current, and the current due to membrane potential changes. Therefore, the HH model also equates the cell membrane to a circuit diagram.

2) IF and LIF models

In 1907, the integrate-and-fire neuron model was proposed by Lapicque (1907). According to the variation of neuronal membrane potential with time in the model, it can be divided into the IF model and the LIF model. The IF model describes the membrane potential of neurons with input current, as shown in Equation 1:


(1) $$\begin{array}{l} C_{m}\frac{dV}{dt}=I \end{array}$$

*C*_*m*_ represents the neuronal membrane capacitance, which determines the rate of change of the membrane potential. *I* represents the neuronal input current. The model is called the leak-free IF model because the neuronal membrane potential is only correlated with the input current. When the current input zero, the membrane potential remains unchanged. Its discrete form is shown in Equation 2:


(2) $$\begin{array}{l} V\left(t\right)=V\left(t-\Delta t\right)+I\left(t\right) \end{array}$$

where Δ*t* is the step length of discrete sampling.

In contrast, the LIF model adds the simulation of neuron voltage leakage. When there is no current input for a certain period of time, the membrane voltage will gradually leak to resting potential, as shown in Equation 3 (citing Equation 1):


(3) $$\begin{array}{l} C_{m}\frac{dV}{dt}=g_{leak}\left(E_{rest}-V\right)+I \end{array}$$

*g*_*leak*_ is the leaky conductance of the neuron. *E*_*rest*_ is the resting potential of the neuron. Neuroscience-related studies have shown that the binding of neurotransmitters to receptors in the postsynaptic membrane primarily affects the electrical conductance of postsynaptic neurons, thereby altering the neuronal membrane potential. So, it is more biologically reasonable to expand the input current *I* in Equation 1 into excitatory and inhibitory currents described by conductance. However, both neurons change to resting potentials directly after activation unable to retain the previous spike.

3) Izhikevich model

In 2003, researcher Eugene M. lzhikevich proposed the lzhikevich model from the perspective of nonlinear dynamical systems (Izhikevich, 2004). It can present the firing behavior of a variety of biological neurons with an arithmetic complexity close to that of the LIF model, as shown in Equation 4:


(4) $$\begin{array}{l} \frac{dV}{dt}=0.04V^{2}+5V+140-U+I \end{array}$$

(5) $$\begin{array}{l} \frac{dU}{dt}=a\left(bV-U\right) \end{array}$$

(6) $$\begin{array}{l} \text{if }V\geq 30mV,\;then=\{\begin{array}{c} v\;=\;c\\ U\;=\;U\;+\;d \end{array} \end{array}$$

In Equations 5 and 6, *U* is an auxiliary variable, adjusted for the parameters *a*, *b*, *c*, and *d*, the lzhikevich model can exhibit a discharge behavior similar to the HH model. But unlike the HH model in which each parameter has a clear physiological meaning (e.g., ion channels, etc.), these parameters no longer have the corresponding properties.

4) AdEx IF model

The AdEx IF model is a modification of the lzhikevich model. However, the AdEx IF model reduces the response speed of the membrane voltage. This results in a gradual decrease in the frequency of pulse delivery from neurons under constant voltage stimulation conditions. We can think of this as a slowing down of the response of neurons that gradually gets “tired” after sending impulses. It is an essential feature of the AdEx IF model that is closer to the HH model in terms of firing behavior results.

The comparison of the above five neuronal models is summarized in Table 1.

**Table 1:** Comparison of neuronal models.

**Neuron models**	**Circuit forms**	**Advantages**	**Defects**
HH	Capacitor resistor circuit	Close to biological neurons, high accuracy	Complex expression, complicated operation
LF	Capacitance	Simple operation	Simple model with memory effect
LIF	Capacitor resistor circuit	Simulate resting state, simple operation	The model is simple and ignores many neurodynamic properties
Izhikevich	**\**	Simulate multiple discharge modes	Low computational efficiency
AdEx lF	**\**	Simulate multiple discharge modes	Reduced pulse firing frequency under constant voltage stimulation

### Neural system information encoding

Neural information encoding consists of two processes: feature extraction and spike sequence generation. In terms of feature extraction, there is no mature theory or algorithm. In terms of spike sequence generation, there are two approaches commonly used by researchers: rate coding (Butts et al., 2007; Panzeri et al., 2010) and temporal coding. Rate coding uses the frequency of spike to express all the information of spike sequences, which cannot effectively describe the fast time-varying perceptual information. Unlike average-rate coding, temporal coding takes into account that precisely timed spike carries valid information. Thus, temporal coding can describe neuronal activity more accurately. Precise spike timing plays an important role in the processing of visual, auditory, and other perceptual information.

1) Rate coding

Rate coding primarily utilizes a stochastic process approach to generate a spike sequences. The response function of a neuron suitable for Poisson coding is consist of a series of spike functions as shown in Equation 7:


(7) $$\begin{array}{l} \rho \left(t\right)=\overset{k}{\underset{i=1}{\sum }}\delta \left(t-t_{i}\right) \end{array}$$

*k* is the number of spikes in a given spike sequence, *t* represents the arrival time of each spike, and *t*_*i*_ denotes the time at which each spike occurs. The unit spike signal is defined as shown in Equation 8:


(8) $$\begin{array}{l} \delta (t)=\{\begin{array}{ll} 0, & if\;t=0\\ 1, & otherwise \end{array} \end{array}$$

The integral is in the form of $\int_{-\infty }^{+\infty }\delta \left(t\right)=1$. The time of neural action potential response is equivalent to the spike release time in a spike sequence. From the pulse function property, the number of pulses within *t*_1_ to *t*_2_ can be calculated by $n=\int_{t_{1}}^{t_{2}}\rho \left(t\right)dt$. Thus, the instantaneous discharge frequency can be defined as the expectation of the neuronal response function. According to the statistical theory of probability, the mean value of the neuronal response function in a short time interval is used as an estimate of the discharge frequency (Koutrouvelis and Canavos, 1999; Adam, 2014; Safiullina, 2016; Shanker, 2017; Allo and Otok, 2019) as shown in Equation 9:


(9) $$\begin{array}{l} r_{M}\left(t\right)=\frac{1}{M}\overset{M}{\underset{j=1}{\sum }}\rho_{j}\left(t\right) \end{array}$$

*r*_*M*_(*t*) is the number of spikes in the entire time window and ρ_*j*_(*t*) is the number of spike responses per neuron. Neither *r*_*M*_(*t*) nor ρ_*j*_(*t*) is a continuous function and only under the condition of infinite time window, a smooth function can be obtained. The rules of encoding are crucial for the mapping between values and spike.

2) Temporal coding

The time-to-first-spike mechanism is generally used in time encoding as the moment of spike issuance, as shown in Equations 10 and 11:


(10) $$\begin{array}{l} T_{s}=T-\frac{T}{I_{\max}}I, \end{array}$$

(11) $$\begin{array}{l} f(t)=\{\begin{array}{ll} 0, & if\;t=T_{s}\\ 1, & otherwise \end{array} \end{array}$$

*I* represents the actual intensity of each image pixel represented in the field of pattern recognition. *I*_max_ represents the maximum value of each pixel intensity. $\frac{T}{I_{\max}}I$ is a time window with a temporal pattern to ensure the pixel intensity value can be converted. *T*_*s*_ is the exact moment of the emitted spike, and a spike sequence will only emit one spike in the time-to-first-spike mechanism.

3) Population coding

Population coding (Leutgeb et al., 2005; Samonds et al., 2006) is a method of representing a stimulus using the joint activity of multiple neurons. Gaussian population coding is the most widely used model for group-skewed coding. In the actual encoding of the SNN, the pixel intensity is set to a real value that is determined by a set of overlapping Gaussian receptive field neurons. The larger the pixel intensity, the larger the value, the shorter the encoding time, and the easier it is for the Gaussian receptive field neurons near the front to generate a spike and form a spike sequence. Let *k* Gaussian receptive field neurons be encoded then, the centers and widths of *k* Gaussian functions are shown in Equation 12:


(12) $$\begin{array}{l} c_{i}=\min\;+\;\frac{\max-\min}{k-2}▪i,\;k=3,4,5......,n \end{array}$$

$$\begin{array}{l} \sigma =\beta ▪\frac{\max-\min}{k-2},k=3,4,5......,n \end{array}$$

This type of encoding is used to encode continuous variables. For example, the population coding method ensures higher accuracy and realism compared to the first two coding methods for coding sound frequencies and joint positions. Due to the characteristics of this encoding method, it can significantly reduce the number of neurons required for the same accuracy. In order to improve the effectiveness of information encoding, the researchers are also trying to introduce different mechanisms in the encoding process (Dennis et al., 2013; Yu et al., 2013).

### Spiking neural networks and learning algorithms

Over the past decades, researchers have drawn inspiration from biological experimental phenomena and findings to explore the theory of synaptic plasticity. Bi and Pope proposed the spike-timing-dependent plasticity (STDP) mechanism and extended it to different spike learning mechanisms (Bi and Poo, 1999; Gjorgjieva et al., 2011), which order of firing, adjusting the strength of neuronal connections.

To solve the supervised learning problem of SNNs, researchers have combined the STDP mechanism with other weight adjustment methods. This mainly contains the gradient descent and Widrow-Hoff rules. Based on gradient descent rules (Shi et al., 1996), Gutig et al. put forward a Tempotron learning algorithm (Gütig and Sompolinsky, 2006). The algorithm updates the synaptic weights according to the combined effect of the pre-synaptic and post-synaptic pulse time difference and the error signal. Ponulak et al. proposed the ReSuMe learning method (Florian, 2012) avoiding the gradient descent algorithm in the gradient solving problem. The SPAN algorithm was proposed in ref. (Mohemmed et al., 2013). The algorithm is similar to ReSuMe, except that it uses a spike convolution transform to convert spikes into analog values before performing operations, which is computationally intensive and can only be learned offline. Based on the gradient descent, an E-Leaning rule is given by the Chronotron algorithm (Victor and Purpura, 1997; van Rossum, 2001). It adjusts the synapse by minimizing an error function that is defined by the difference in the pulse sequence of the target and actual output. In a comparison of the single-spike output of neurons (e.g., Tempotron) and multi-spike output (e.g., ReSuMe), it was found that the multi-spike output of neurons can greatly improve classification accuracy and learning capacity (Gardner and Gruning, 2014; Giitig, 2014). Therefore, the use of neurons with multi-spike input–output mapping as computational units is the basis for designing efficient and large learning capacity SNNs. Although multi-spike input–output mapping can be implemented, it is only applicable to single-layer SNNs. In the literature (Ghosh-Dastidar and Adeli, 2009; McKennoch et al., 2009; Sporea and Gruning, 2013; Xu et al., 2013), researchers have tried to study algorithms applicable to multilayer SNNs. However, the algorithms for multilayer SNNs are still limited by the current algorithms, and the research on multilayer SNNs is still in its initial stage.

Since the training algorithm for SNNs is less mature, some researchers have proposed algorithms to convert traditional ANNs into SNNs. A deep ANN-based neural network is trained by a comparable mature ANN training algorithm, then, transformed into an SNN by firing rate encoding (Diehl et al., 2015), thus avoiding the difficulty of training SNNs directly. Based on this conversion mechanism, HRL Labs researchers (Cao et al., 2014) converted a Convolutional Neural Network (CNN) (Liu X. et al., 2021) to a Spiking CNN with recognition accuracy close to that of a CNN on the commonly used object recognition test set Neovision2 with CIFAR-10. There is another SNN architecture called liquid state machine (LSM) (Maass et al., 2002), which can also avoid direct training of SNNs. As long as the SNN is large enough, it can theoretically achieve any complex input classification task. Since LSMs are regression neural networks, this confers on them the ability to memorize and can effectively handle the analysis of temporal information. New Zealand researcher Nikola Kasabov proposed the NeuCube system (Kasabov et al., 2016) architecture based on the basic idea of LSM for temporal and spatial information processing. In the training phase, NeuCube uses STDP, a halo-inspired genetic algorithm, etc. to train the SNN. In the operation phase, the parameters of the SNN and the output layer classification algorithm are also dynamically changing, which gives the NeuCube system a strong adaptive capability.

## Brain-like computing chips

### Darwin chip

**Figure 3:**
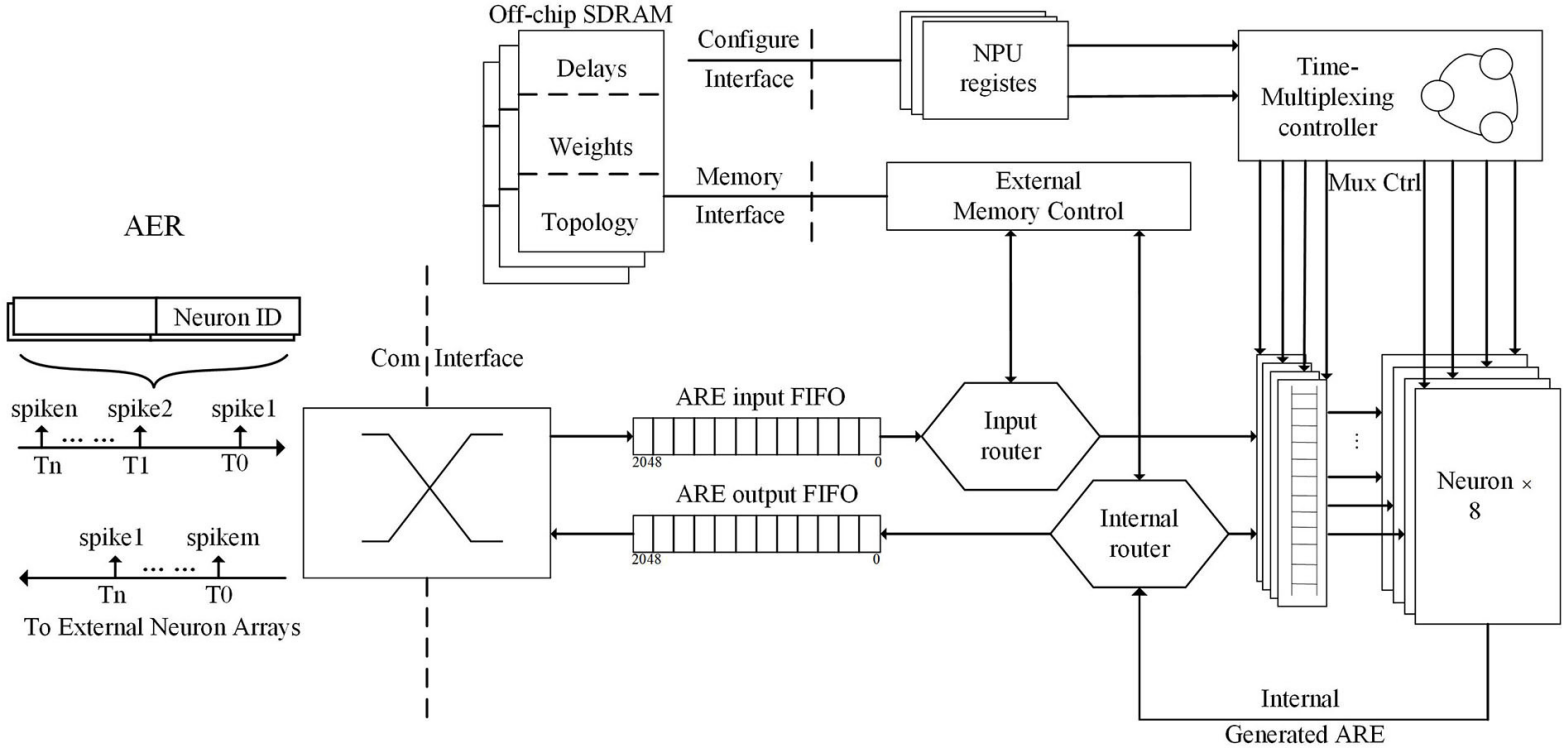
Overall microarchitecture of the Darwin Neural Processing Unit (NPU) and the process of processing AER packages and outputting them.

Figure 3 shows the overall microstructure of the Darwin Neural Processing Unit (NPU) (Ma et al., 2017). The Address-event representation (AER) is the format that represents the input and output spike information encoded. AER packet contains the neuronal ID that generates the spike and the timestamp when the spike is generated, which can represent each spike. The NPU, driven by the input AER packets, works in an event-triggered approach works. Spike routers translate spikes into weighted latency information by accessing storage and SDRAM (Stankovic and Milenkovic, 2015; Goossens et al., 2016; Ecco and Ernst, 2017; Li et al., 2017; Garrido and Pirsch, 2020; Benchehida et al., 2022).

The execution steps of the AER connection runtime are shown below:

#### Time calibration

The NPU works on an event-driven basis. When the FIFO receives a peak, it sent an AER packet to the NPU. The timestamp of the AER packet will be checked by the NPU. The AER packet will enter the peak routing process, if it matches the current time, or, it will go to the neuron state update process.

#### Input spike routing

Each AER packet's input spike consists of the timestamp and the source (presynaptic) neuron ID. It is used to find the target (postsynaptic) neuron ID and synaptic properties, containing weights and delays stored in the off-chip DRAM.

#### Synaptic delay management

Each synapse has an independently configurable delay parameter. The parameter defines the delay from the generation of the presynaptic neuronal spikes to the reception of the postsynaptic neuronal spikes. Each entry of the weights and queues has the intermediate result of the weights and is sent to the neuron after a certain delay.

#### Neuron state update

Each neuron updates its state. First, the neuron receives the biological neuronal current state being updated from the local state memory. Then, it receives the sum of the weights of the current step from the weights and queue. If an output spike generates, it will be sent to the spike router as an AER packet.

#### Internal spike routing

It is similar to the process of input spike routing.

Darwin Chip's NPU is an SNN-based neuromorphic co-processor, while it still is a single-chip system, for now, the standard communication interface defined by the AER format allows expanding to multi-chip distributed systems (Nejad, 2020; Cui et al., 2021; Hao et al., 2021; Ding et al., 2022) with AER bus connections in the future. NPU, as a processing element in a network-on-a-chip (NoC) architecture200, can use the AER format for input and output peaking to scale the SNN's size of the chip to potentially millions of neurons, not just thousands of neurons.

### Tianjic chip

**Figure 4:**
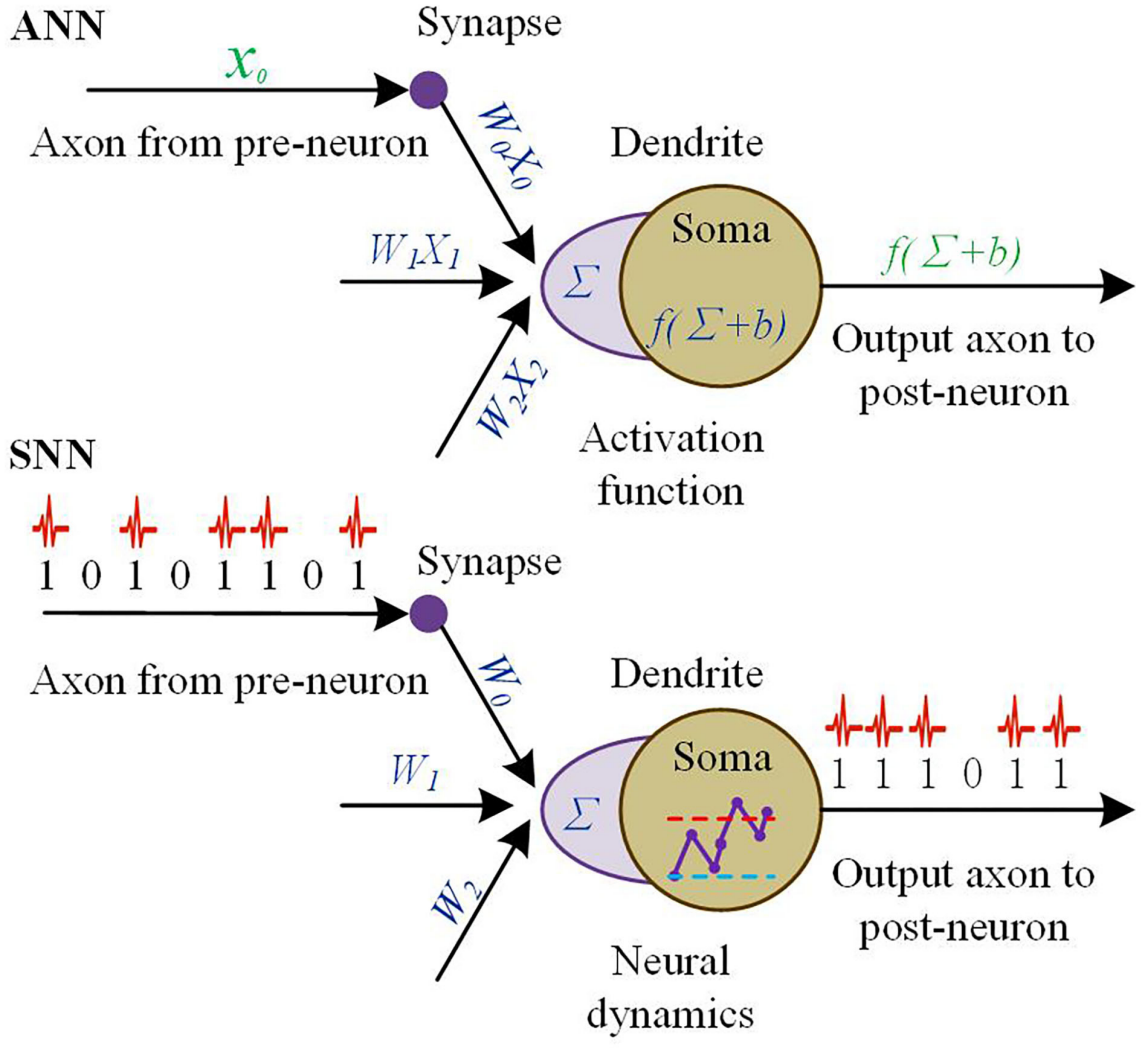
The unified functional core (Fcore) of the Tianjic chip consists of four main components: axons, dendrites, soma, and router.

The Tianjic team is committed to create a brain-like computing chip that has the advantages of both traditional computer and neuromorphic computation. To this end, the researchers designed the unified functional core (Fcore) of the Tianjic chip (Pei et al., 2019), which consists of four main components as follows: axons, dendrites, soma, and router. Figure 4 shows the architecture of the Tianjic chip.

#### Axon block

The Tianjic team sets a small buffer memory to record the historical spikes in SNN (Yang Z. et al., 2020; Agebure et al., 2021; Liu F. et al., 2021; Syed et al., 2021; Das et al., 2022; Mao et al., 2022) mode. The buffer memory can support reconfigurable peak collection durations and bit access *via* shift operations.

#### Dendritic blocks

Membrane potential integration of SNN mode and MACs (multiply-and-accumulate) of ANN mode use the same calculator together in order to reunify the level of abstraction of SNNs and ANNs during processing. In detail, MAC units are used to multiply and accumulate in ANN mode. In SNN mode, there is a bypassing mechanism that can skip multiplication to reduce energy under a time window of length one.

#### Soma

In SNN mode, the Soma is reconfigurable in order to have peak resetting, deterministic, potential storage, probabilistic fire, and threshold comparison. In ANN mode, fixed or adaptive leakage of the potential value can reduce the leakage function of the membrane potential.

**Figure 5:**
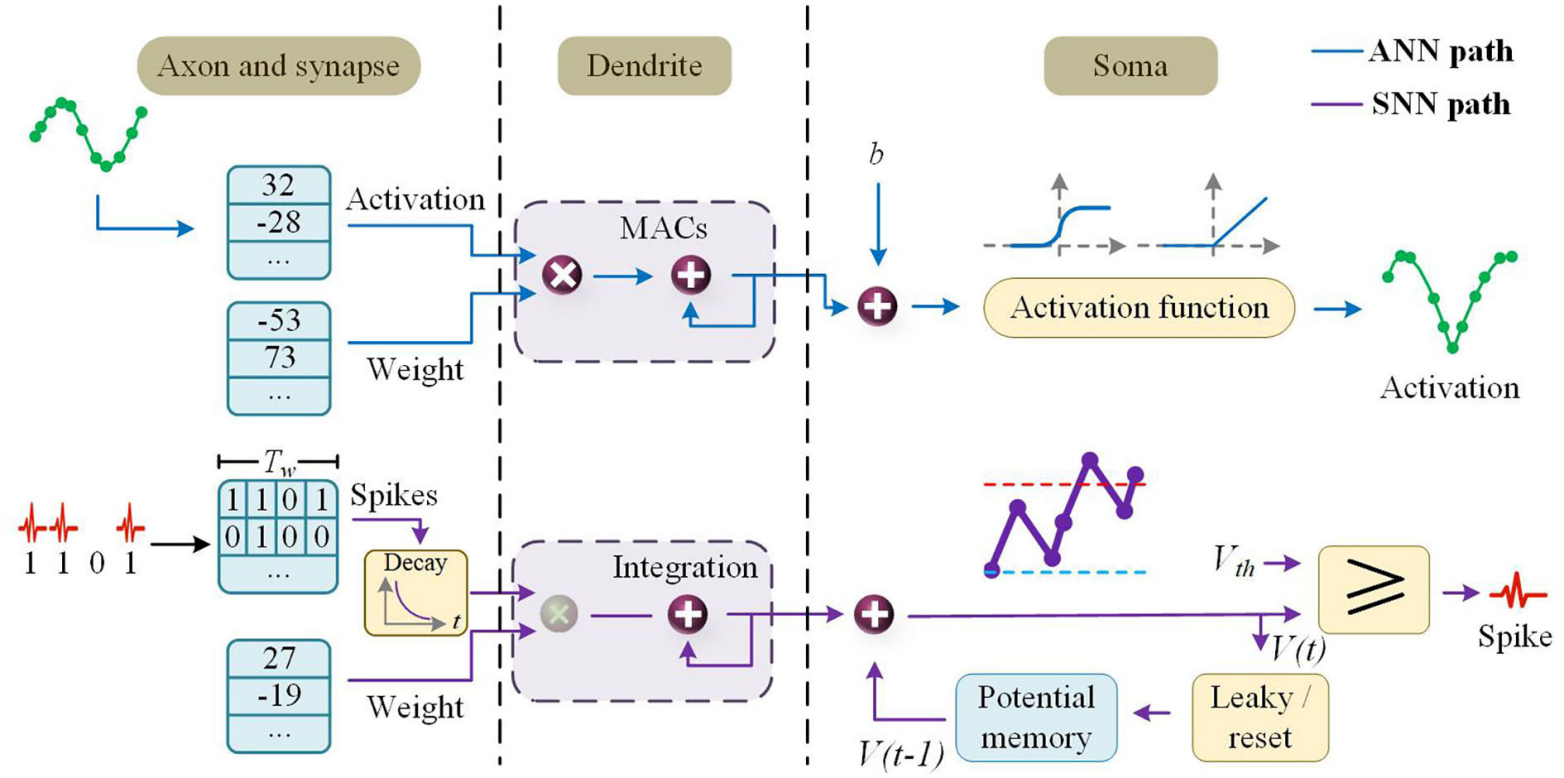
Design of the Tianjic chip and its specific processing flow in ANN and SNN mode.

To transmit information between neurons, a router receives and sends information, which is responsible for the transmission and conversion of information between cell bodies and axons. The Design of Tianjic chip is shown in Figure 5.

**Figure 6:**
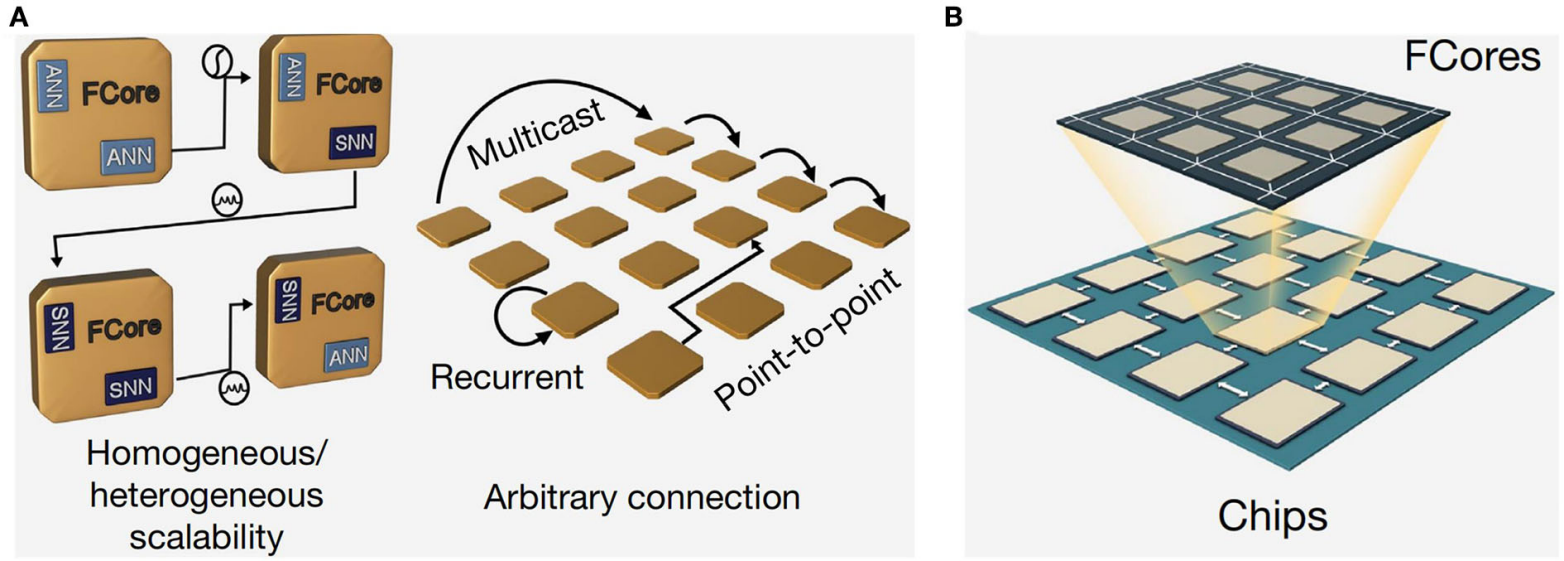
Arrangement format of Fcores on the Tianjic chip. **(A)** Reconfigurable routing tables of the routers of FCore have the ability of arbitrary connection topologies. **(B)** The arrangement of Fcore on the chip.

In order to support parallel processing of large networks or multiple networks, the chip equipped with a multi-core architecture (Chai et al., 2007; Chaparro et al., 2007; Yu et al., 2014; Grassia et al., 2018; Kiyoyama et al., 2021; Kimura et al., 2022; Zhenghao et al., 2022) can perform seamless communication at the same time. The FCores of the chip, shown in Figure 6, arrange in a two-dimensional (2D) grid. Reconfigurable routing tables of the routers of FCore have the ability of arbitrary connection topologies.

### TrueNorth

**Figure 7:**
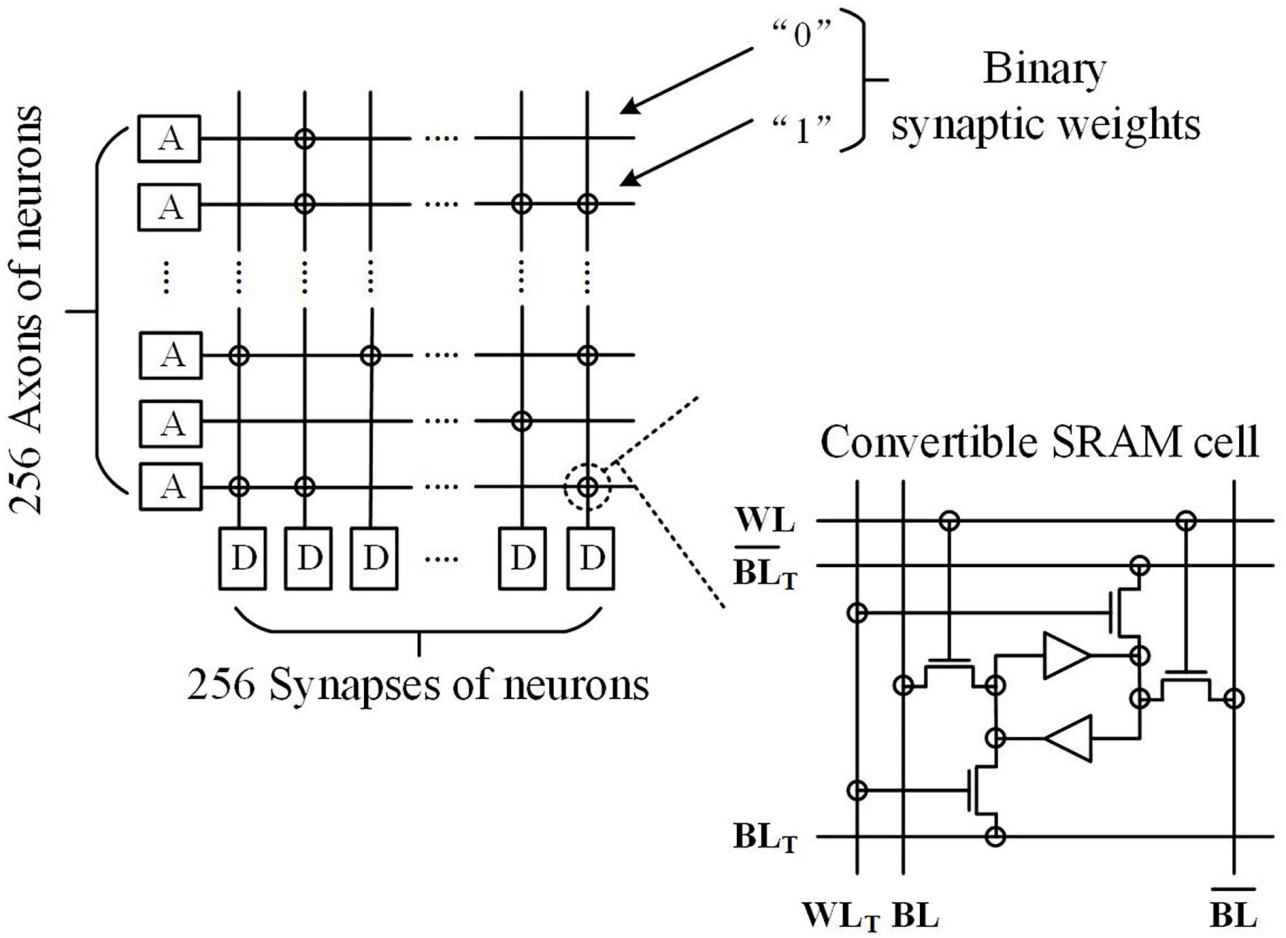
Neuromorphic synaptic apparatus spiking neural network core block.

IBM started from the level of neuronal composition and principles to build a brain-like computing chip by mimicking the brain structure and performing neural simulation with the help of the spike signal conduction process (Birkhoff, 1940). Starting from neuroscience, the neuromorphic synaptic nuclei are used as the basic building blocks of the entire network (Service, 2014; Wang and Hua, 2016). The designers of TrueNorth consider neurons as the main arithmetic unit. The neuron receives and integrates the “1” or “0” pulse signal and issues instructions based on that signal. Then output the instructions to other neurons through the synapses at the connections between neurons (Abramsky and Tzevelekos, 2010; Russo, 2010). It is shown in Figure 7.

The data transmission is implemented in two stages: first, the transmission data between core blocks are passed along the *x*-direction and then along the *y*-direction until it reaches the target core block. Then, the information is transmitted within the core block, where in the same core block it first passes through presynaptic axons (horizontal lines), cross-aligned synapses (binary junctions), and finally, to the input of the postsynaptic neuron (longitudinal lines).

**Figure 8:**
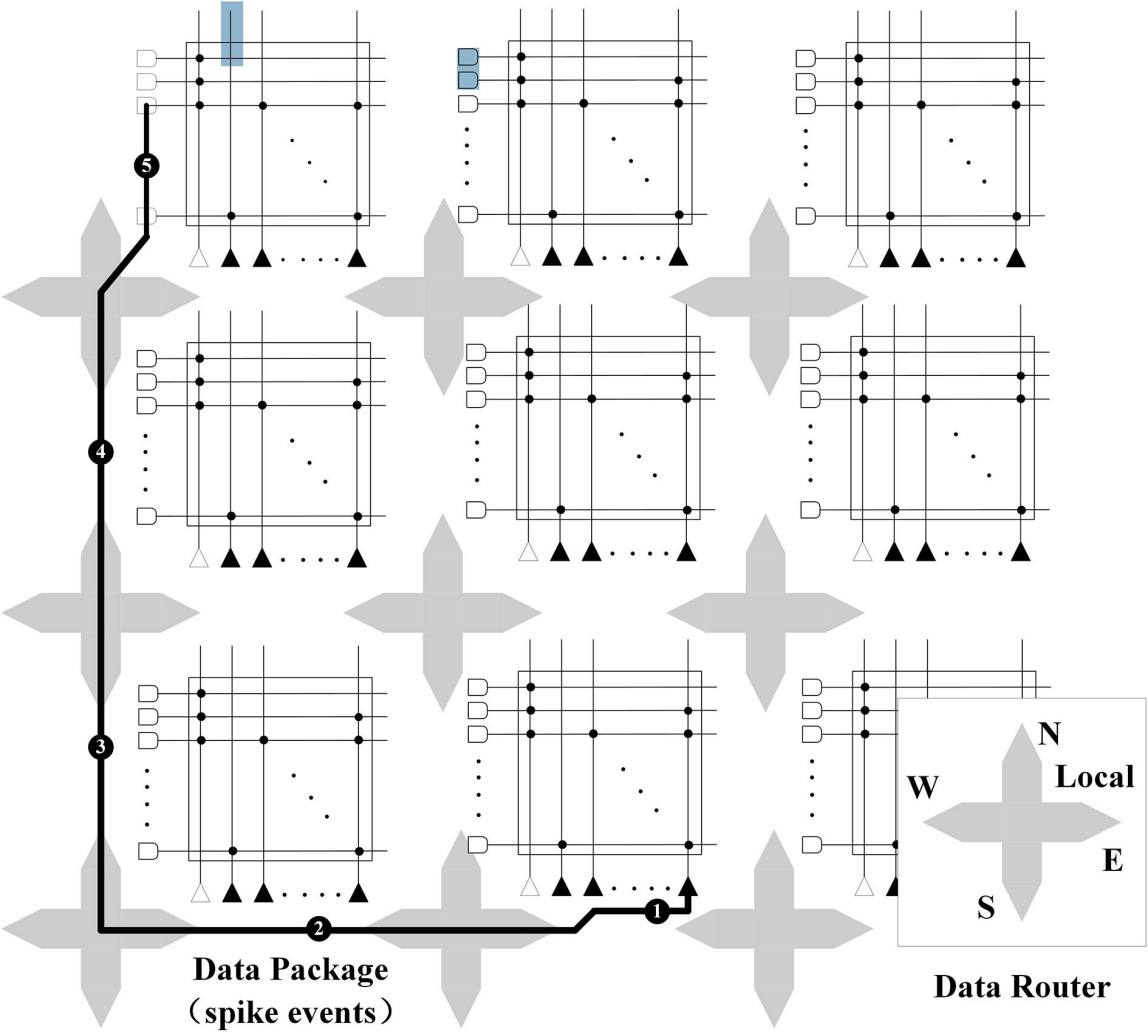
The propagation of router data between core blocks.

When a neuron on a nucleus block is excited, it first searches local memory for the axon delay value and the destination address, and then encodes this information into a data packet. If the destination neuron and the source neuron are in the same nucleus block, the local channel in the router is used to transmit the data, otherwise, they will use the channel in the other direction. To prevent the limitation caused by the excessive number of nucleus blocks, a combined decentralized structure is used at the four edges of the network. When leaving the core block, the spike leaving the network is marked with upward (east–west direction) and column (north–south direction). When entering the core block, the spikes sharing the link input are propagated to the corresponding row or column using the marking information, as shown in Figure 8. The global synchronization clock is 1 Khz, which ensures that the one-to-one hardware and software corresponds exactly to the core block operating in parallel (Hermeline, 2007).

### Neurogrid

**Figure 9:**
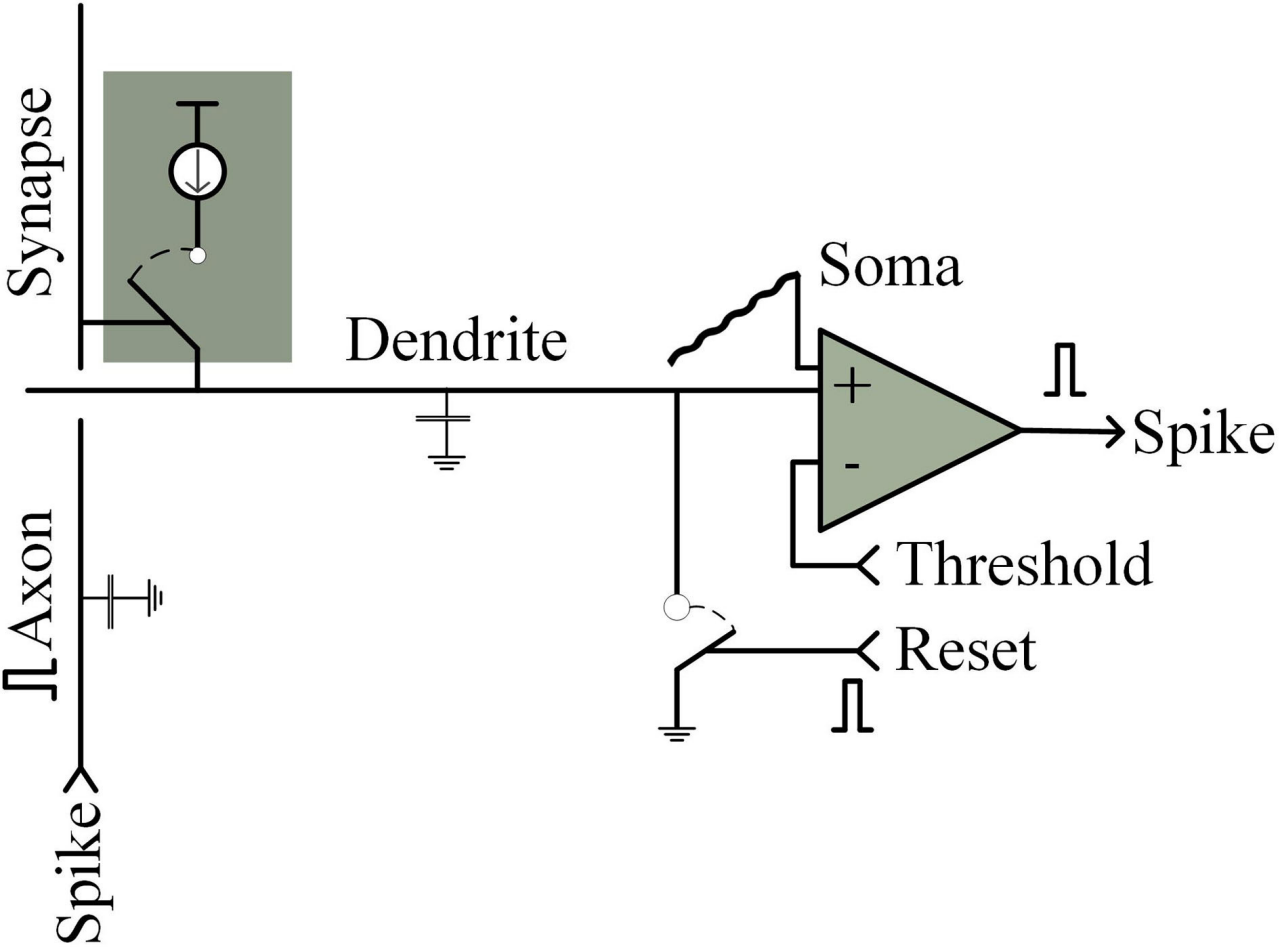
Analog silicon neurons implementation.

The Neurogrid chip (Benjamin et al., 2014) consists of axonal, synaptic, dendritic, and cytosolic parts. Neurogrid chips are available in four structures: fully dedicated (FD) (Boahen et al., 1989; Sivilotti et al., 1990), shared axons (SA) (Sivilotti, 1991; Mahowald, 1994; Boahen, 2000), shared synapses (SS) (Hammerstrom, 1990; Yasunaga et al., 1990), and shared dendrites (SD) (Merolla and Boahen, 2003; Choi et al., 2005). The four elements that make up the chip, axon, synapse, dendrite, and soma can be classified according to the architecture and the implementation. As shown in Figure 9, in the analog implementation, a switched current source, a comparator, a wire, and another wire mode are the four elements, respectively (Mead, 1989). A vertical wire (axon) instrumentation charge (synapse) is inserted into a horizontal wire (dendrite), and the charge will be integrated by the capacitance of dendrite. The generated voltage is compared with the threshold by the comparator (cell) and the comparator triggers an output peak if the voltage exceeds a threshold. After that, the capacitor will be discharged (reset) and starts a new cycle.

**Figure 10:**
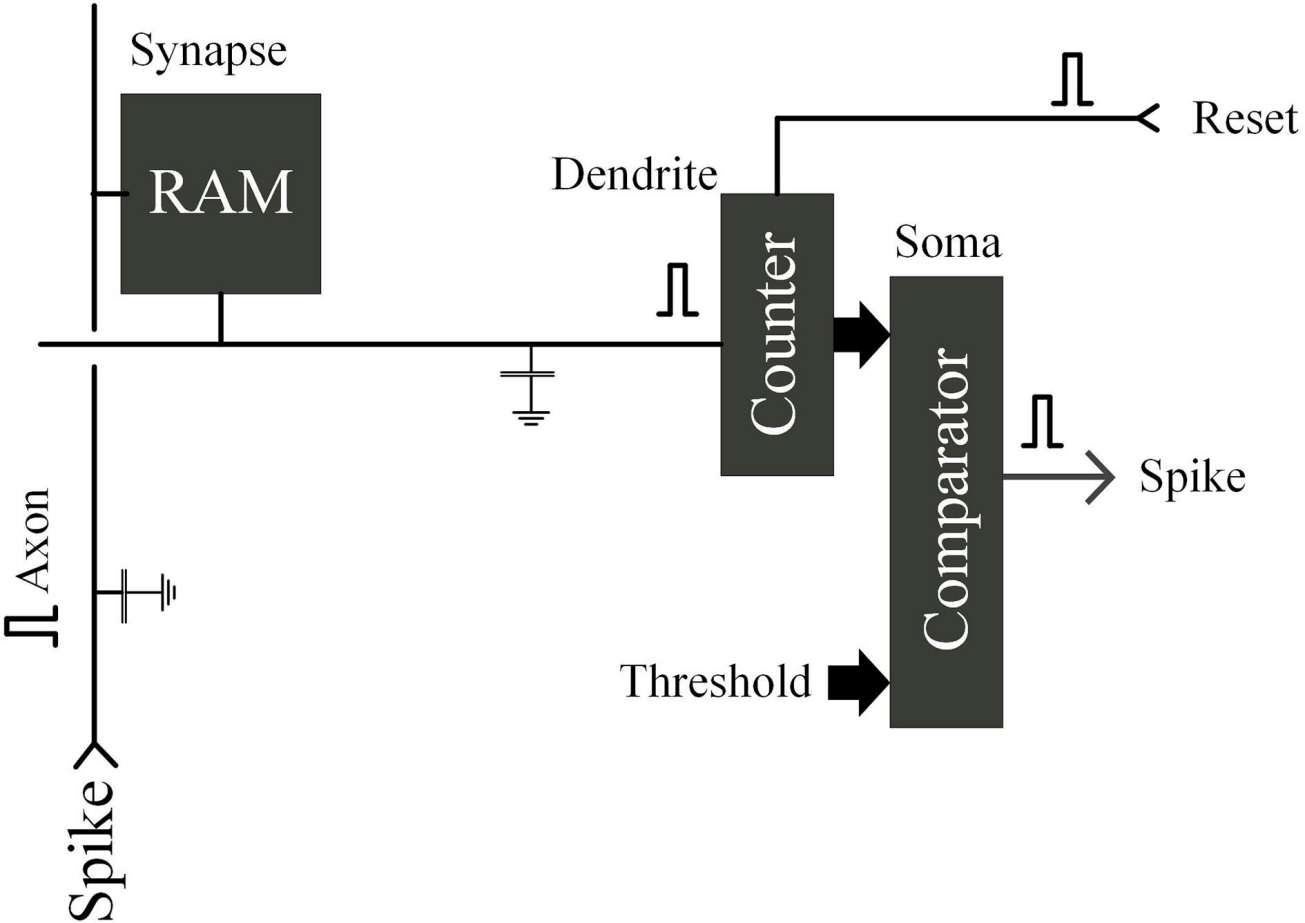
Schematic diagram of analog neuron with cycle counting structure.

In the simplest all-digital implementation, the switching current source is replaced by a bit unit. Functions of axonal and dendritic as word and bit lines are integrated and compared, respectively, digitally: In a loop, a binary 1 is read from the synapse, triggered by the axon, the counter increments (dendrite), and the counter's (cell) output is compared with the threshold digitally stored. During the threshold, the counter will be reset and start a new cycle if a peak is triggered. The process is shown in Figure 10.

Spikes of a neuron are sent from its array through a transmitter, passed through a router to the parents and two children of its neural core, and passed through a receiver to the receivers. All these digital circuits are event driven and their logic synthesizes vat only when a spike event occurs, following Martin's asynchronous circuit (Martin, 1989; Martin and Nystrom, 2006). The chip has a transmitter and a receiver. The receiver sends multiple peaks to one row and then, the transmitter sends multiple peaks from the row. The address of the common row and the unique column of these peaks will be communicated sequentially. This design facilitates an increase in throughput during communication.

### BrainScaleS-2

The BrainScaleS team released two versions of the BrainScaleS chip design in 2020, and here in this article, we present the BrainScaleS-2 chip (Schemmel et al., 2003; Grübl et al., 2020). The architecture of BrainScaleS-2 depends on a tight interaction of analog circuit blocks and digital circuit blocks. Due to the main intended function of the digital processor core, it is referred to as the plasticity processing unit (PPU). The analog core serves as the main neuromorphic component and includes synaptic and neuronal circuits (Aamir et al., 2016, 2018), PPU interfaces, analog parameter memory, and all event-related interface components.

**Figure 11:**
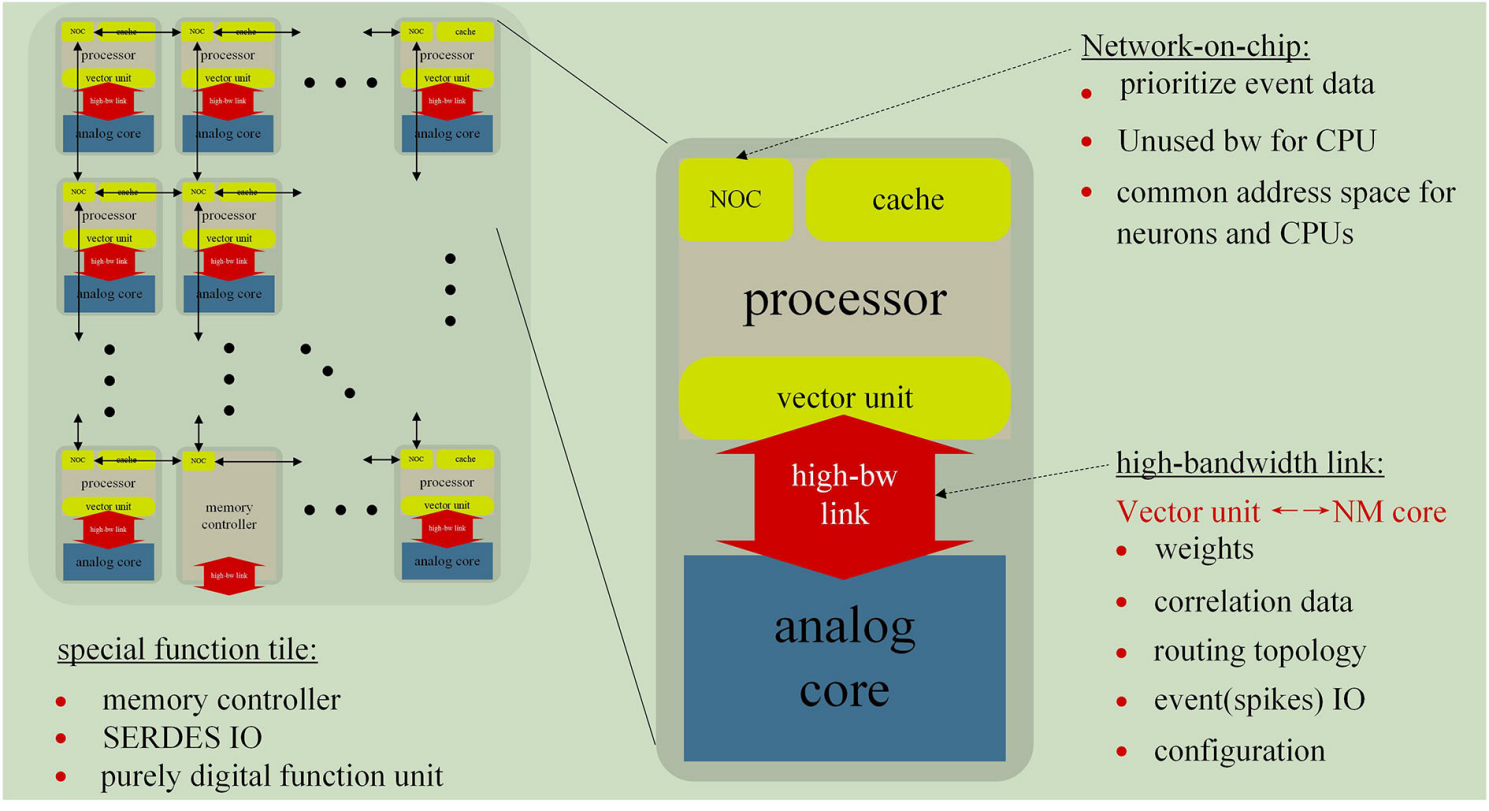
BBS chip structure.

There is a digital plasticity processor in the BSS-2ASIC (Friedmann et al., 2016). This microprocessor, which is specialized in highly parallel single instruction multiple data (SIMD), has an additional layer of the capability of modeling. Figure 11 shows the architecture.

**Figure 12:**
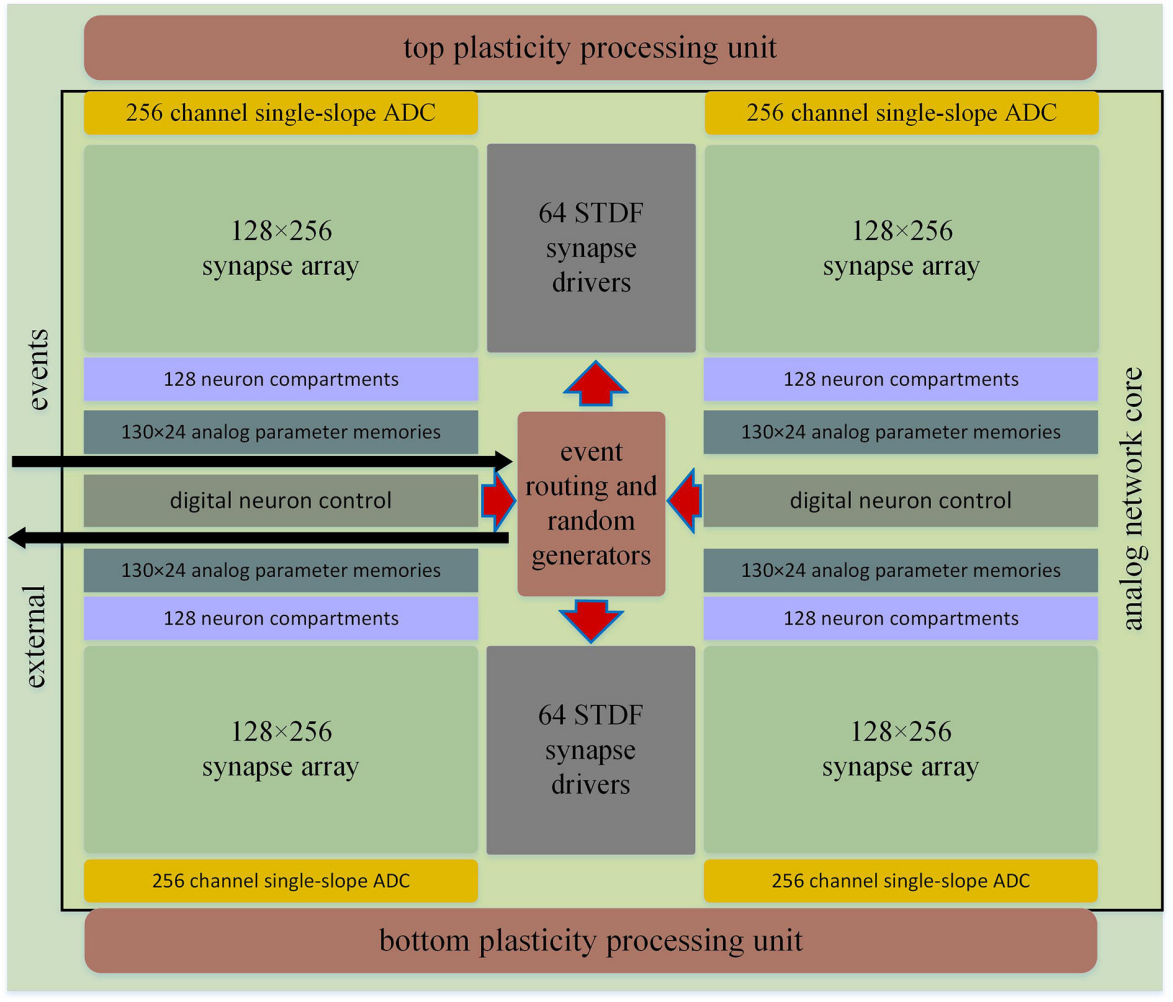
Block diagram of the Analog Network Core (ANNCORE).

The PPU is an embedded microprocessor core with the SIMD units. The unit and simulation core are together optimized for computing plasticity rules (Friedmann et al., 2016). In the current architecture of BSS-2, the same simulation core can be shared by two PPUs. This makes the neuronal circuits to the most efficient arrangement in the center of the simulation core. Figure 12 shows the individual functional blocks in the ring core.

#### Synaptic arrays

In order to make the vertical and horizontal lines that through the subarrays as short as possible, synapses are divided into four blocks of equal size. This reduces their parasitic capacitance (Friedmann et al., 2016; Aamir et al., 2018). Each synaptic array resembles a static memory block and each synapse has 16 memory cells. Two PPUs are connected to the static memory interface of the two adjacent synaptic arrays using a fully parallel connection with 8 x 256 data lines.

#### Neuronal compartment circuits

Four rows of neuronal compartment circuits are placed at the synaptic blocks' edge. Each pair of dendritic input lines of the neuronal compartment connects with 256 synapses. Neuron chambers implement the AdEx neuron model.

#### Analog parameter memories

There is a row of simulation parameters storage between each row of neurons. There are 24 simulated values and another 48 global parameters stored in these capacitive memories for each neuron. These parameters can automatically refresh with the reception of values from the storage block.

#### Digital neuron control

The digital neuron control block can be shared by two rows of neurons, which synchronize neural events to a 125-MHz digital system clock and serializes them to the digital output bus.

#### Synaptic drives with short-term plasticity

Presynaptic events of the array are input by synaptic drivers. They contain circuits that simulate the simplified Tsodys–Markram model (Tsodyks and Markram, 1997; Schemmel et al., 2007) of short-term plasticity. Synaptic drivers can handle single- or multi-valued input signals.

#### Random event generator

The random generator is fed directly into the synaptic array *via* the synaptic driver through the synaptic driver, which greatly reduces the use of external bandwidth when using the random model (Pfeil et al., 2015; Jordan et al., 2019).

#### Correlation analog to digital converters (ADCs)

SIMD units of PPU arrange the location of the top and bottom edges of the ring core. Analog data from the synaptic array and given analog signals from the neurons are converted into the digital representation required by the PPU by column-parallel ADCs.

The Table 2 summarizes the above representative domestic and international brain-like computing projects and chips or hardware stations.

**Table 2:** Brain-like chip summarization and comparison.

**Project & organization**	**Manufacturing process**	**Number of neurons**	**Number of synapses**	**Neuronal models**	**Learning algorithms**	**Advantages**	**Defects**
Darwin, Zhejiang University	180 nm COMS	2,048	4,194,304	LIF	\	Highly configurable	Single chip, small scale
Tianjic, Tsinghua University	28 nm COMS	40,000	100,000,000	LIF	STDP	Heterogeneous fusion	\
TrueNorth	28 nm COMS	1,000,000	256,000,000	LIF	\	Highly configurable	Off-chip learning only
Neurogrid	l80 nm COMS	1,048,576	hundreds of millions	QIF	\	High throughput	No plasticity
BrainScaleS-2	65 nm COMS	196,608	50,331,648	AdEx IF	STDP	Mixed plasticity rule	Does not demonstrate the ability to handle practical tasks

## Brain-like computing application

### Brain cognition principle

The main advantages of neuromorphic computing over traditional methods are energy efficiency, speed of execution, robustness to local failures, and learning ability. Currently, neuroscientific knowledge of the human brain is only superficial and the development of neuromorphic computing is not guided by theory. Researchers hope to refine models and theories by using brain-like computing for partial simulations of brain function and structure (Casali et al., 2019; Rizza et al., 2021).

In 2018, Rosanna Migliore et al. (2018) used a unified data-driven modeling workflow to explore the physiological variability of channel density in hippocampal CA1 pyramidal cells and interneurons. In 2019, Alice Geminiani et al. (2019) optimized extended generalized leaky integrals and excitation (E-GLIF) neurons. In 2020, Paolo Migliore et al. (2018) designed new recurrent spiking neural networks (RSNNs) in the brain based on voltage-dependent learning rules. Their model can generate theoretical predictions for experimental validation.

Brain-like computing can help neuroscience understand the human brain more deeply and parse its structure (Amunts et al., 2016; Dobs et al., 2022). After understanding enough about the operation mechanism of the human brain, we can act directly on the human brain to improve thinking ability and solve the currently untreatable brain diseases. What is more, it can make the human intelligence level break through to new heights.

### Medical health

The application of brain-like computing in medical field mainly relies on the development and application of brain–computer interface (Mudgal et al., 2020; Huang D. et al., 2022). It is reflected in the following four aspects: monitoring, improvement, replacement, and enhancement.

Monitoring means that the brain–computer interface system completes the real-time monitoring and measurement of the human nervous system state (Mikołajewska and Mikołajewski, 2014; Shiotani et al., 2016; Olaronke et al., 2018; Sengupta et al., 2020). It can help grade consciousness in patients in a deep coma and measure the state of neural pathways in patients with visual/auditory impairment. Improvement means that we can provide recovery training for ADHD, stroke, epilepsy, and other conditions (Cheng et al., 2020). After doctors detect abnormal neuronal discharges through brain–computer interface technology, they can apply the appropriate electrical stimulation to the brain to suppress seizures. “replacement” is primarily for patients who have lost some function due to injury or disease. For example, people who have lost the ability to speak or speech can express themselves through a brain–computer interface (Ramakuri et al., 2019; Czech, 2021); groups of people with severe motor disabilities can communicate what they are thinking in their heads through a brain–computer interface system. “enhancement” refers to the strengthening of brain functions by implanting chips into the brain (Kotchetkov et al., 2010). For example, it enhances memory and helps a person to call mechanical devices directly.

### Intelligence education

The education and development of children is an important issue of social concern. But the research on children's development and psychological problems has been conducted only through dialog and observation. Brain-like computing research hopes to directly observe the corresponding brain waves and decoding of brain activity.

In the “Brain Science and Brain-like Research” project guidelines, the state mentions the use of brain-like technology to study the mental health of children and adolescents, including the interaction between emotional problems and cognitive abilities and their brain mechanisms, the development of screening tools and early warning systems for emotional problems in children and adolescents by combining machine learning (Dwyer et al., 2018; Yang J. et al., 2020; Du et al., 2021; Paton and Tiffin, 2022) and other means, and encouraging the integration of medicine and education. Eventually, we will develop psychological intervention and regulation tools for children and adolescents' emotional problems, and establish a platform for monitoring and intervening in children and adolescents' psychological crises based on multi-level systems, such as schools and medical care.

### Intelligent transportation

Nowadays, self-driving cars have many sensors, including radar, infrared, camera, GPS, and so on, but the car still does not have the ability to make the right decision like a human. Humans only need to use vision and hearing among their senses to ensure the safe driving of the vehicle. The human brain has powerful synchronous and asynchronous processing capabilities for reasonable scheduling, and human eye recognition is more accurate than all current camera recognition.

Inspired by the way neurons in the biological retina transmit information, Mahowald and Mead proposed in the early 1990s an asynchronous signal transmission method called AER (Tsodyks et al., 1998; Service, 2014). When a pixel in a pixel array occurs an “event,” the position of this pixel is output with the “event.” Based on this principle, the Dynamic Vision Sensor (DVS) (Amunts et al., 2016) was developed at the University of Zurich, Switzerland, to detect changes in the brightness of pixels in an image. The low bandwidth of DVS gives it a natural advantage in the field of robot vision, and work has been done to use it in autonomous walking vehicles and autonomous vehicles. Dr. Shoushun Chen of Nanyang Technological University, Singapore, developed an asynchronous sensing chip with a temporal sensitivity of 25 nanoseconds (Schemmel et al., 2003, 2010; Scholze et al., 2012). The brain-like cochlea (Scholze et al., 2012) is a brain-like auditory sensor based on a similar principle that can be used for sound recognition and localization. The results of all these studies will accelerate the implementation of autonomous driving and ensure the safety of the autonomous driving process.

### Military applications

Brain-like chips have the technical potential for ultra-low-power consumption, massively parallel computing, and real-time information processing. It has unique advantages in military application scenarios, especially in conditions with strong constraints on performance, speed, and power consumption. It can be used for ultra-low latency dynamic visual recognition against military targets in the sky, and the formation of a cognitive supercomputer to achieve rapid processing of massive amounts of data (Czech, 2021). In addition, brain-like computing can be used for intelligent gaming confrontation and decision-making in the future battlefield.

The ultra-low-power consumption, ultra-low latency, real-time high-speed dynamic visual recognition, tracking technology, and sensor information processing technology of the brain-like chip is a key technology at the strategic level of national defense science and technology. Especially the ultra-low latency real-time high-speed dynamic visual recognition technology has an extremely important role in the field of high-speed dynamic recognition. In 2014, the U.S. Air Force signed a contract with IBM to make high-altitude flying targets efficient and low powered through brain-like computing. The U.S. Air Force Research Laboratory began developing a brain-like supercomputer using IBM's True North brain-like chip in June 2017. In the following year, the laboratory released the world's largest neuromorphic supercomputer, the Blue Jay. The computer can simultaneously simulate 64 million biological neurons and 16 billion biological synapses, and power consumption is only 40 watts, 100 times lower than traditional supercomputers. They plan to demonstrate an airborne target recognition application developed by the Blue Jay in 2019. By 2024, they will enable real-time analysis of 10 times more big data than current global Internet traffic. This turns the big data that constrain the next generation of warplanes from a problem to a resource and greatly shortens the development cycle of defense technology and engineering.

## Challenges

### Novel observation and simultaneous modulation techniques for brain activity face challenges

Brain observation and regulation technology are an important technical means to understand the input, transmission, and output mechanism of brain information and is also the core technical support to understand, simulate, and enhance the brain. Although various *in vivo* means of acquiring and modulating brain neural information by MRI, optical/optical genetic imaging, and other technologies are becoming more abundant and rapidly developed, the following problems still exist in current research: single observation mode and modulation means, partial observation information, lack of knowledge of brain function, inability to synchronize brain modulation, and observation.

#### The mathematical principles and computational models of brain information processing are not well developed

Neuroscientists have a clearer understanding of the single neuron model, the principles of partial neural loop information transfer, and the mechanisms of primary perceptual functions. But the global information processing in the brain, especially the understanding of higher cognitive functions, is still very sketchy (Aimone, 2021). To build a computational model that can explain the brain information processing process and perform cognitive tasks, we must understand the mathematical principles and brain information processing.

#### Immature hardware processes for brain-like computing

The use of hardware to simulate brain-like computational processes still faces an important challenge in terms of brain-like architectures, devices, and chips. On the one hand, CMOS and other traditional processes have encountered bottlenecks in on-chip storage density and power consumption (Chauhan, 2019), while new nano-devices still have outstanding problems, such as poor process stability and difficulty in scaling. Brain-like materials and devices require new technologies to break through current bottlenecks (Chen L. et al., 2021; Chen T. et al., 2021; Wang et al., 2021; Zhang et al., 2021). On the other hand, brain-like systems require tens of billions of neurons to work together. However, the existing brain-like chip is difficult to achieve large-scale interconnected integration of neurons and efficient real-time transmission of neuronal pulse information under the constraints of limited hardware resources and limited energy consumption.

#### The efficiency of the existing human brain thinking answers needs to be improved urgently

Due to the complexity of the brain and the great difference between brain and machine, it brings poor robustness of brain signal acquisition, low efficiency of brain–machine interaction, lack of brain intelligence intervention means, high requirement of brain area intervention targets, and difficulty of fusion system construction. Given the complementary nature of machine intelligence and human intelligence, how to efficiently interpret the information transmitted by the human brain, realize the interconnection of biological intelligence and machine intelligence, integrate their respective strengths, and create intelligent forms with stronger performance are the main challenges of brain-like research (Guo and Yang, 2022).

## Prospects

### Brain-like computing model

The study of brain-like computing models (Voutsas et al., 2005) is an important foundation of brain-like computing, which determines the upper limit of neuromorphic computing from the bottom, mainly divided into neuron models, neural network models, and their learning methods. We can look forward to the development of brain-like computational models in the following directions: studying the dynamic coding mechanisms of biological neurons and neural networks, establishing efficient spike coding theories and models with biological rationality, multimodal coordination, and joint representation at multiple spatial and temporal scales; studying and exploring the coordination mechanisms of multisynaptic plasticity, the mechanisms of cross-scale learning plasticity, and the global plasticity mechanisms of biological neural networks; establish efficient learning theories and algorithms for deep SNNs to realize intelligent learning, reasoning, and decision-making under multi-cognitive collaborative tasks of brain-like; to study mathematical descriptions of different levels of brain organization and continuous learning strategies under multi-temporal neural computational scales to realize rapid association, transfer, and storage of multimodal information.

### Neuromorphic devices

The current development of artificial neuromorphic devices mainly includes two technical routes. One is based on the traditional mature CMOS technology of SRAM or DRAM build (Asghar et al., 2021), and the prototype device is volatile in terms of information storage; the other is built based on non-volatile Flexible FLASH devices or new memory devices and new materials (Feng et al., 2021; He et al., 2021). Non-volatile neuromorphic devices are memristors with artificial neuromorphic characteristics and unique nonlinear properties that have become new basic information processing units that mimic biological neurons and synapses (Yang et al., 2013; Prezioso et al., 2015). In future inquiries, we need to clarify some basic questions: in neural operations, which level is needed to simulate the neural properties of organisms, and which functions are primary in neuromorphic operations. These issues are critical to the implementation of neural computing.

### Neuromorphic computing chips

Artificial neural network chips have made progress in practical applications, whereas pulsed neural network chips are still in the exploratory application stage. Future research on neuromorphic chips can try to study neuromorphic computing chips from several different directions, such as architecture, operation method, and peripheral circuit technology of neuromorphic component arrays suitable for convolutional and matrix operations, hardware description, mapping scheme of neural network algorithm to new neuromorphic component arrays, compensation algorithm and circuit compensation method for various non-ideal factors of new neuromorphic components, and data routing method between arrays.

### Neuromorphic computing supporting system

However, the results are not satisfactory in practical applications. For example, the efficiency of online learning is much lower than the speed of neural computing, and the efficiency and accuracy of SNN learning are not as good as traditional ANN. We can study the high-efficiency deployment of neural network learning training algorithms, the compensation method of learning performance loss during the process of computing efficiency improvement, and carry out flow verification of prototype prototypes; we can build a large-scale brain-like computing chip simulation platform with online learning functions and demonstrate a variety of online brain-like chip-based learning applications. Develop the potential of neuromorphic computing in terms of platforms, systems, applications, and algorithms.

At present, brain-like computing technology is still a certain distance away from being formally put into industrial production (Zou et al., 2021), but it will certainly be one of the important points of contention between various countries and enterprises in the next 10 years. So, this is an opportunity for all researchers, and whether it can be truly applied in production life depends on the researchers' key research results in certain aspects. We hope that we researchers will achieve a technological breakthrough to bring brain-like to life as soon as possible.

## Author contributions

WO: conception and design of study. CZ: participated in the literature collection, collation of the article, and was responsible for the second and third revision of the article. SX: acquisition of data. WH: analysis and interpretation of data. QZ: revising the manuscript critically for important intellectual content. All authors contributed to the article and approved the submitted version.

## Funding

This work was supported in part by the Hainan Provincial Natural Science Foundation of China (621RC508), Henan Key Laboratory of Network Cryptography Technology (Grant/Award Number: LNCT2021-A16), and the Science Project of Hainan University (KYQD(ZR)-21075).

## Conflict of interest

The authors declare that the research was conducted in the absence of any commercial or financial relationships that could be construed as a potential conflict of interest.

## Publisher's note

All claims expressed in this article are solely those of the authors and do not necessarily represent those of their affiliated organizations, or those of the publisher, the editors and the reviewers. Any product that may be evaluated in this article, or claim that may be made by its manufacturer, is not guaranteed or endorsed by the publisher.
